# Social Media News Headlines and Their Influence on Well-Being: Emotional States, Emotion Regulation, and Resilience

**DOI:** 10.3390/ejihpe14060109

**Published:** 2024-06-05

**Authors:** Marilena Mousoulidou, Loukia Taxitari, Andri Christodoulou

**Affiliations:** Department of Psychology, Neapolis University Pafos, Paphos 8042, Cyprus; l.taxitari@nup.ac.cy (L.T.); andri.christodoulou@nup.ac.cy (A.C.)

**Keywords:** headlines, social media, news, well-being, emotional states, emotion regulation, resilience

## Abstract

Today, many individuals read the daily news from social media platforms. Research has shown that news with negative valence might influence the well-being of individuals. Existing research that examined the impact of headlines on individuals’ well-being has primarily focused on examining the positive or negative polarity of words used in the headlines. In the present study, we adopt a different approach and ask participants to categorize the headlines themselves based on the emotions they experienced while reading them and how their choice impacts their well-being. A total of 306 participants were presented with 40 headlines from main news sites that were considered popular based on the number of public reactions. Participants had to rate their emotional experience of the headlines following five emotional states (i.e., happiness, anger, sadness, fear, and interest). Emotion regulation strategies and resilience were also measured. In line with our hypotheses, we found that participants reported experiencing negative emotions more intensively while reading the headlines. Emotion regulation was not found to influence the emotional states of individuals, whereas resilience did. These findings highlight that individuals can experience heightened emotions without reading the entire news story. This effect was observed regardless of the headline’s emotional valence (i.e., positive, negative, or neutral). Furthermore, our study highlights the critical role of interest as a factor in news consumption. Interest significantly affects individuals’ engagement and reactions to headlines, regardless of valence. The findings underscore the complex interplay between headline content and reader engagement and stress the need for further research into how headlines are presented to protect individuals from potential emotional costs.

## 1. Introduction

Social media has seamlessly integrated into our daily lives, emerging as the predominant digital activity globally. According to Statistica [1], it constitutes 59% of the global population and is embraced by 92.7% of all internet users [2]. As of July 2023, there are over 4.88 billion social media users worldwide [3], which is expected to reach almost 6 billion by 2027 [1]. The role of social media in disseminating news and information is pivotal, shaping the way we consume and engage with current events. Social media platforms serve as dynamic and instantaneous conduits for the distribution of news, allowing information to reach a vast and diverse audience in real time. Individuals spend significant time on popular social media sites such as Facebook, YouTube, and Instagram. The rise of the Internet in the 21st Century changed traditional news consumption. Whereas in the past, people predominately focused on printed newspapers for everyday news, now, there is a consistent increase in people reading their news via digital means. With the advent of technology in the early 2000s, digital media has exemplified and broadened the way in which we gain information. Digital media refers to any information that is electronically stored, accessed, and transmitted, and it is profoundly different from traditional media, which refers to printed work. Digital media has transformed how information is consumed by making it readily available, interactive, and visually attractive [4]. Digital news content has become a common feature on the start pages of browsers and social networking sites and may be distributed via push notifications. News contact via such features is not generally initiated by users, occurring instead incidentally as a by-product of other activities. This kind of news contact is termed Incidental News Exposure [5] and appears to be more widespread in social media [6] (see [7] for a recent review). 

News agencies incorporated social media to distribute their news articles and embraced social media to connect with their audiences through sharing and recommending news content [8]. This transformation of news dissemination has changed the traditional media landscape, enabling individuals to share and access information with unprecedented speed and accessibility. Individuals can now search and find news and information that is important and relevant to them and also interact with people regarding this information. They can share the news, discuss it with others, and even contribute to the news [8]. The significant role of social media in distributing news has been particularly observed during the COVID-19 pandemic. Research that was conducted during that time showed that social media was one of the most important channels to disseminate information [9,10,11]. One of the main reasons was the speed at which information was distributed in social media, which enabled individuals to stay up to date with news, recommendations, and policies regarding an evolving virus [11]. 

There is a growing number of available information channels and sources in social media, ranging from well-established news channels to news channels that are only available online, making social media a competitive environment. For something to be considered news, it needs to be current, factual, and objectively presented information that is valuable and important to people [12,13,14]. However, the information that is prioritized in social media, newspaper headlines, and online websites does not always provide “important and valuable” news items [12,13]. Readers do not always read information that they find useful and relevant to them [14]. It appears that what counts as news is not based on valuable information but rather information that will gain more attention from the users. Quite often, individuals read news articles not because they are interested in the subject but because the articles were shared on social media and are, therefore, more readily available [8]. Moreover, reading news from social media raises the possibility of readers encountering fake news or false information. In fact, research has shown that online sites and social media have become the major sources from which to spread misleading and false information [15], as they facilitate quick and widespread sharing [16]. This issue is closely related to polarization, as the media often emphasizes divergent viewpoints to the extent that it fosters distinct and opposing segments within society (see [17] for a review). Fake news refers to mainstream news content that has been fabricated or is extremely inaccurate but is presented in a way that aesthetically resembles actual legitimate news [18], and false information referring to misinformation presented as verified fact [19] is particularly problematic. Professional persuaders exploit this phenomenon, among others, for profit through online advertising [20], using it as clickbait. Clickbait refers to online news headlines designed in such a way as to entice users into clicking on a link [21]. It is apparent that fake news can easily act as clickbait, using strategies such as sensationalism, suspense, and stylistic formats that make the headlines more tempting and interesting for readers [21]. Nevertheless, not all news that is shared on social media and online sites is fake news or clickbait. News that is produced by reputable organizations is more likely to be true [22,23].

The flow of news on social media creates a continuous battle for news agencies trying to grasp their audiences’ attention by making their stories appear urgent, relevant, and sometimes unusual. Headlines are important in this respect since they are created in such a way as to maximize interest and grab attention [24,25,26]. A headline allows individuals to scan a large number of articles and obtain abbreviated news updates. They provide a summary of the article’s main idea, enabling individuals to decide which articles are of interest and should be read [24,25,26]. Their role in news communication is so important that it has been argued that many newspaper readers spend more time scanning headlines than reading the full stories [24]. This appears to be a strategy that enables readers to gain information with minimal cognitive effort [24]. Headlines, therefore, need to be creative to attract readers’ attention and make them want to read the full article [27]. They need to be presented in a way that even if the majority of people do not consider them interesting, it would justify spending energy and time reading them [13]. Research has shown that creative headlines are preferred even when this makes the headline more confusing, less informative, and longer [25]. 

The Emotions-as-Frames Model [28,29], a significant framework for understanding the role of emotion in online news consumption, proposes an interplay between emotional content and information processing. This model suggests that the emotional tone of a news story acts as a “frame”, influencing how and what information is perceived, processed, and remembered and how behavioral responses are shaped. By emphasizing certain details and minimizing others, the model implies that emotions can shape the interpretation of information, thus framing the cognitive and perceptual field. Specifically, emotionally framed news may increase the probability of readers searching for associated information or suppressing further exploration of certain topics. For example, emotions such as hope and anger may encourage and reinforce the exploration of greater information, whereas fear and anxiety may result in avoidance behavior [29]. This model is supported by research that showed that different emotions lead to varying levels of information-seeking [28]. 

Building on the insight from the Emotion-of-Frames Model [28,29], it becomes evident that news headlines designed to evoke strong emotional responses are particularly effective. These headlines often use sensationalism as a strategy to maximize attention and reader engagement. Sensationalism, a common strategy for enhancing the appeal and urgency of headlines, involves highlighting the emotional or dramatic aspects of the story to provoke the audience’s emotions or attention [30]. This type of coverage often emphasizes topics like disasters, crime, sex, and celebrity affairs, using dramatic language, narratives, or graphic imagery that portray events in news headlines as being more personally relevant, interesting, and extraordinary than they truly are [13,31]. The aim of such coverage is to trigger emotional reactions in the reader [32]. Sensationalistic tactics in headlines appear to be the preferred strategy for digital news coverage [30,33,34,35,36,37]. Note here that sensationalism also relates to the concept of “post-truth”, which prevails in political journalism. Although it is beyond the scope of this research, it is worth mentioning that this phenomenon refers to circumstances where truth and objective facts become relative and often subordinate to the power of personal belief and emotions [38,39].

Sensationalism’s impact is profound, especially when paired with the use of loaded emotional language in headlines. Research has shown that such headlines are not only more memorable but also more striking, capturing readers’ attention effectively [40]. Emotions play a pivotal role in this interaction, affecting not only individuals’ attention to news topics but also their overall engagement on social media (see [41] for a review). Emotions are complex multicomponent psychophysiological processes that comprise bodily/physiological reactions, cognitive processes, and the subjective experience of emotion known as affect [42,43]. They are often viewed as varying in valence between positive and negative. Positive emotions are more complex than simple sensations and reflect a desirable or pleasurable emotional response to the environment, such as happiness, contentment, and excitement [43,44]. Negative emotions, on the contrary, are viewed as reflecting a general feeling of distress, such as anger, sadness, and disgust [43]. Note here that Gasper et al. [45] recently suggested that there is also a neutral affect, which refers to feeling indifferent or nothing in particular about a certain situation. Interestingly, despite the potential for headlines ranging in valence, the literature suggests that news headlines frequently emphasize negative valence rather than neutral or positive [33,34,36,37]. This focus likely stems from findings suggesting that individuals tend to pay more attention to negative news topics, like war, crime, and disasters [37], leading to an increase in coverage of such topics over the last decade [46]. This focus intensified especially during the pandemic, because uncertainty and fear prevailed [47,48]. For instance, research conducted by Aslam et al. [47] using sentiment analysis, an approach that enables researchers to identify the positive or negative polarity of words [47,49], demonstrated that over half of the 141,208 news headlines that were analyzed were related to negative sentiments. Similar findings were obtained from another study that analyzed the posts and comments of people during COVID-19 and found that negative sentiments were increasing as cases of COVID-19 were increasing [50]. 

This emphasis on negative framing aligns with the findings of Kim and Cameron [51], who examined how emotional responses impact individuals’ information processing. In their experiment, participants were presented with a fictitious news story regarding a corporate crisis that was either anger-inducing or sadness-inducing, and their response to the story was measured. Findings showed that participants demonstrated greater processing on sadness-inducing frames as compared to anger-producing frames. Additionally, the media’s initial framing impacted the evaluation of the effectiveness of corporate messages. These findings suggest that the emotional content of media coverage not only captures attention but also impacts a person’s assessment of a situation. News coverage that emphasizes dramatic narratives or distressing images can significantly impact public perception and behavior, making the strategic use of sensationalism a powerful tool in news reporting.

The relationship between news consumption (positive or negative) and well-being is well documented in the literature [34,52,53,54,55,56]. Such research suggested that repeated consumption of negative news might influence individuals’ well-being and emotional states. For instance, in a longitudinal study, de Hoog and Verboon [34] asked participants to report five times a day, for ten days, the instances they perceived news reports, the type and valence of the news they perceived, and their emotions and appraisals regarding the news. They found that when participants perceived daily news as negative, they reported more negative affect and less positive affect, a finding that was also reported in other studies [53]. Other research showed that positive news stories make individuals feel good [55]. Particularly, McIntyre and Gibson [55] found that participants who read positive news stories reported higher positive emotions, such as enjoyment, than those who read negative news stories. Clearly, therefore, there is an interaction between news valence and well-being.

A set of strategies that individuals can use to control their mood states is emotion regulation. Emotion regulation is a term that refers to a set of processes that individuals use to influence which emotions they experience, when they experience them, how intensively they experience them, and how they express these emotions [57,58,59]. Even though there are many different strategies that individuals can use to regulate their emotions, two basic strategies are Reappraisal and Suppression [57]. Reappraisal is a strategy that enables individuals to modify how an emotion-generative situation is evaluated, whereas suppression concerns inhibiting or hiding ongoing emotion-expressive behavior [57,59]. Emotion regulation strategies, therefore, can be used by individuals to control the possible negative impact of news headlines. Resilience is another strategy that is linked to the experience of emotions. Resilience is a coping strategy that enables individuals to successfully adapt or recover after the occurrence of an adverse life event [60,61]. Consequently, resilience is typically triggered by such events, and it is not surprising that research on resilience often focuses on its role during critical periods. Existing research that examined resilience in the context of news exposure has shown that the valence of news (positive or negative) can influence individuals’ resilience levels [48,62]. For instance, Giri and Maurya [48] examined individuals’ resilience and emotions when exposed to positive or negative news about COVID-19. They found that resilience and positive emotions were significantly decreased when individuals were exposed to negative news, while resilience and positive emotions were significantly higher when individuals were exposed to positive news [48]. Similarly, in a recent study, Malecki et al. [62] found that negative media experiences about the Ukraine war were associated with lower resilience levels. Hence, it is clear that resilience is impacted by the valence of news. 

Even though the literature suggests that news headlines might impact individuals’ emotions, only a few studies directly examined the emotions individuals experience while reading news headlines. Most of these studies have focused on examining the positive or negative polarity of words used in news headlines via sentiment analysis [47,49,50]. However, for a more thorough understanding of the way individuals feel when they read news headlines, it is imperative to have the participants themselves report the emotions they experience when reading these headlines. Given the lack of such research, the present study aims to examine the impact of news headlines on individuals’ well-being by focusing on the emotions that individuals experience while reading headlines that are popular on social media news sites. To our knowledge, there is no other study in the literature that examined Cyprus and Greece in this respect. In particular, in the current study, participants are asked to categorize the headlines based on five emotional states: (a) happiness, (b) anger, (c) sadness, (d) fear, and (e) interest. These ratings aim to capture the participants’ emotional experience while reading the headlines. Each of these emotions is characterized by distinct appraisal components and physiological expressions. Happiness is a high-arousal emotion that is characterized by feelings of joy and contentment, often triggered by events perceived as beneficial or satisfying [63,64,65]. Anger arises from instances perceived as frustrating or unjust, leading to displeasure or annoyance and a predisposition to confront the source of anger [63,64,65]. Sadness is a typical response to failure or loss, involving a sense of displeasure and despair and a desire for the lost object [63,64,65]. Fear is elicited by perceived threats to security or safety that cause anxiety and protective behaviors [63,64,65]. Lastly, interest elicits increased attention, curiosity, and exploratory behavior [63,64,65,66]. It is considered a newly explored emotion in the emotional literature and has been found to conflate with happiness [66]. These emotions are selected for two main reasons: (a) they are recognized in the literature as fundamental to human experience, indicating their universal relevance and applicability across different settings [67,68], and (b) they correspond to the emoticons actively used by individuals on social media (especially Facebook) to express their reactions to the content [69,70]. This correspondence makes them particularly relevant for analyzing how individuals process and react to social media news headlines. In addition, since emotion regulation and resilience might influence the experience of emotions, these two variables are also examined. Based on the aims and the existing literature, the following hypotheses are formulated:

**Hypothesis** **1** (**H1).**  *Participants’ ratings of the emotional valence (i.e., positive, negative, and neutral) depicted in the headlines are expected to differ. Based on the available literature, more negative than positive emotions are expected to be evoked.*

**Hypothesis** **2** **(H2).**  *There will be associations between the five emotional states (i.e., happiness, anger, fear, sadness, and interest) and emotion regulation strategies. Since these strategies are used to control the possible negative impact of headlines, it is hypothesized that individuals using these strategies will be less likely to experience emotions with negative valence, like anger, sadness, or fear, after reading the headlines.*

**Hypothesis** **3** **(H3).**  *Associations between the five emotional states (i.e., happiness, anger, fear, sadness, and interest) and resilience are anticipated. Previous findings suggest links between the experience of emotions and resilience levels. Although our study focuses on overall resilience rather than resilience specific to critical periods, we expect to find associations between the emotional responses to reading headlines and overall resilience levels.*

## 2. Materials and Methods

### 2.1. Sample

This study’s final sample consisted of 306 individuals (female = 233, 71.14%) with a mean age of 37.26 (range = 17–83 years). All participants were monolingual native Greek speakers from mainland Greece or Cyprus and residing in one of the two countries. One participant was excluded from the final analysis because they were bilingual speakers of Greek and Russian.

### 2.2. Measures

*Demographic information*: Data were collected on participants’ age, gender, native language, and place of residence. 

*Emotion Regulation*: The Greek version of the Emotion Regulation Questionnaire (ERQ) [59,71] was used to examine the emotion regulation strategies used by the participants. The ERQ is a 10-item questionnaire rated on a 7-point Likert scale ranging from 1 (strongly disagree) to 7 (strongly agree). The questionnaire examines two emotion regulation strategies: cognitive reappraisal (e.g., “When I want to feel less negative emotion, I change the way I’m thinking about the situation”) and suppression (e.g., “I control my emotions by not expressing them”). The ERQ indicated good internal consistency with a Cronbach’s alpha value of 0.80. 

*Resilience*: The Brief Resilience Scale (BRS) [72,73] was developed to gauge individuals’ perceived capacity to rebound or recover from stress. It is a six-item scale designed to evaluate a unified concept of resilience, encompassing both positively and negatively phrased items. Scores on the BRS can vary within a range from 1 (indicating low resilience) to 5 (indicating high resilience). Respondents provide ratings on a 5-point Likert scale, ranging from 1 (strongly disagree) to 5 (strongly agree). A higher mean BRS score indicates a greater level of resilience. The items’ internal reliability for the current sample was good, with a Cronbach’s alpha value of 0.82. 

*Newspaper Headlines*: A tailored survey was crafted to gauge emotional responses to headlines. Participants were asked to rate the intensity of the emotion caused by each headline on a scale of 1 (not at all) to 7 (very intense) for the emotions happiness, sadness, anger, fear, and interest. This examination aimed to identify patterns and trends in emotional responses to headlines across these top-ranking news sources. The study delved into an analysis of the five news sites that are most commonly used in the Greek-speaking world, as determined by the number of followers on social platforms. Facebook and Twitter were chosen as the most popular platforms for the dissemination of news articles. Forty headlines were included in the final sample based on their popularity on either Facebook or Twitter, as determined by the number of public reactions, with a minimum threshold of 1000 reactions (range: 1000–14,000 reactions), irrespective of what kind of reactions they were. Reactions were not related to specific emotions, as the emoticons accompanying posts could not be directly associated in a one-to-one relationship with the emotions in this study. Notably, headlines related to politics, religion, and sports were deliberately excluded from the study. This exclusion aimed to narrow the focus and avoid potential biases associated with these specific content categories. Refer to Appendix A for a list of the 40 headlines used in the current study.

### 2.3. Procedure

After approval by the Cyprus National Bioethics Committee, the questionnaires were set up in electronic form through Google Forms. Participant recruitment included advertisements through social networks (Facebook and Instagram), social chat applications (Viber and WhatsApp), and private emails to friends, colleagues, organized groups, and students. 

Before completing the online questionnaires, participants were informed about their right to withdraw from the survey and maintain their anonymity. They were given contact details in case they had questions or needed more information about the study. Informed consent to participate was secured by pressing the “consent” button before proceeding to complete the questionnaires.

The survey remained online for two weeks, and before recruitment stopped, a sample number of 300 participants was secured. Participants were recruited online and had no contact at all with the researchers. Demographic information was collected, but data were fully anonymized. Since we had not included any demographic questions in our hypotheses, we did not control for these factors in our sample (for example, gender). Any participant who wished could freely take part in the study, provided they adhered to the inclusion criteria.

### 2.4. Data Processing and Measures

Upon the completion of data collection, several factors were extracted from the questionnaires. The BRS was used to extract a BRS total score for each participant, indicating their resilience levels, as well as categorical BRS levels, indicating low, middle, and high resilience level, calculated based on the instructions provided by the creators of the questionnaire, with low resilience scores from 1 to 2.99, medium resilience scores from 3 to 4.3, and high resilience scores from 4.31 to 5. Similarly, the ERQ was used to calculate two factors, (a) cognitive reappraisal and (b) emotional suppression, for each participant, indicating their emotion regulation levels. Additionally, and similarly to the BRS group, a cognitive reappraisal level and an emotion suppression level were created based on the quartiles calculated in the sample, resulting in a three-level variable (low, middle, and high Levels).

Since the headlines were in the Greek language, the demographics questionnaire was used to ensure that all participants were native speakers of Greek, either from Greece or Cyprus. Gender and Age were used as factors in subsequent analyses. 

Finally, the ratings of the emotional states for each of the 40 headlines were used to create total emotion scores for the emotional states (i.e., happiness, anger, sadness, fear, and interest). Additionally, the newspaper headlines were categorized post hoc into three emotional valence levels: positive, neutral, and negative headlines. The categorization was based on the ratings of the emotional states received by participants. Quartiles were calculated for all emotional states, and the 4th quartile (more than 75%) was used to categorize emotional states into groups: headlines within the 4th quartile for happiness were categorized as positive; headlines within the 4th quartile for anger, sadness, or fear were categorized as negative; and the rest were categorized as neutral. Interest was not considered in this categorization since it was unclear whether it was related to positive or negative emotional states. It was ensured that headlines categorized as positive did not fall within the 4th quartile of any negative emotional states (sadness, anger, or fear) and vice versa. Negative headlines did not fall in the 4th quartile for happiness. One title was excluded because it fell in the 4th quartile for both positive and negative emotions. This resulted in 16 neutral headlines, 14 negative headlines, and 9 positive headlines.

### 2.5. Analysis

Preliminary analyses included the calculation of descriptive statistics for the different emotional states. Univariate ANOVAs were run with rating as the dependent variable and emotional states and gender/age as the fixed factors. 

The main analysis included Repeated Measures ANCOVA models with emotional states (happiness, sadness, anger, sadness, fear, and interest) and emotional valence level (positive, neutral, and negative) as within-subject factors, and BRS, cognitive reappraisal, and emotion suppression level as fixed factors. Univariate ANOVAs and LSD post hoc tests further studied the main effects and interactions between the different factors. 

## 3. Results

A preliminary analysis focused on the ratings of the different emotional states by participants, along with tests for age and gender effects. Table 1 indicates the overall emotion ratings for the five emotional states used in the current study. As it is clear, sadness received the highest mean rating, followed by interest, fear, and anger. Happiness received the lowest mean rating. These findings supported Hypothesis 1.

**Figure 1:**
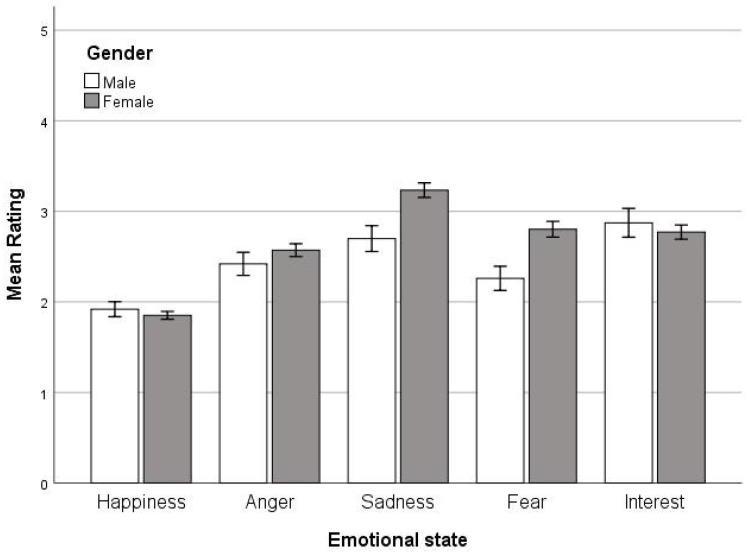
Mean ratings for the five emotional states based on gender, with error bars representing +/−1SD.

A Univariate ANOVA with rating as the dependent variable and emotional states and gender as the fixed factors revealed high main effects for both emotional states (*F*(4, 1470) = 30.71, *p* < 0.001) and gender (*F*(1, 1470) = 9.95, *p* < 0.01), as well as an interaction between the two (*F*(4, 1470) = 4.37, *p* < 0.01). Post hoc analyses showed that all emotional states were significantly different from each other (ps < 0.01), except fear and anger, and fear and interest. The main effect of gender resulted in females (M = 2.65, SD = 1.19) having higher ratings than men (M = 2.43, SD = 1.15) overall. The significant interaction between emotional states and gender suggested that men and women behaved differently across emotional states, and therefore, Univariate ANOVAs were run for each emotional state separately and gender as a fixed factor. There was a significant effect of gender only for sadness (*F*(1, 294) = 10.66, *p* < 0.01) and fear (*F*(1, 294) = 10.03, *p* < 0.01), but not for other emotional states. Gender was not included as a factor in subsequent analyses because the number of participants from each gender and each other group (BRS and ERQ levels) was not enough for meaningful analyses to be conducted. Figure 1 shows the mean ratings for the five emotional states based on gender.

Additionally, all emotional states and age were entered into Pearson r correlations to test their relationships. Age was not significantly related to any emotional state. Emotional states, on the other hand, were all significantly correlated with each other, as shown in Table 2, with medium to high correlations (rs = 0.44–0.88). Age was not considered in subsequent analyses. 

To examine Hypothesis 2, the effect of emotion regulation on emotional states evoked by newspaper headlines was examined. A Repeated Measures ANOVA was conducted, with rating as a dependent variable, emotional states (happiness, anger, sadness, fear, and interest) and emotional valence level (neutral, positive, and negative) as within-subjects factors, and suppression/reappraisal levels as a fixed factor. These replicated the main effect of emotional states (suppression: *F*(4, 307) = 146.60, *p* < 0.001/reappraisal: *F*(4, 285) = 140.22, *p* < 0.001) and emotional valence level (suppression: *F*(2, 303) = 378.63, *p* < 0.001/reappraisal: *F*(2, 287) = 354.05, *p* < 0.001), as well as the interaction between them (suppression: *F*(8, 297) = 115.94, *p* < 0.001/reappraisal: *F*(8, 281) = 107.17, *p* < 0.001), but showed no other main effects or interactions. Therefore, Hypothesis 2 was not supported.

**Figure 2:**
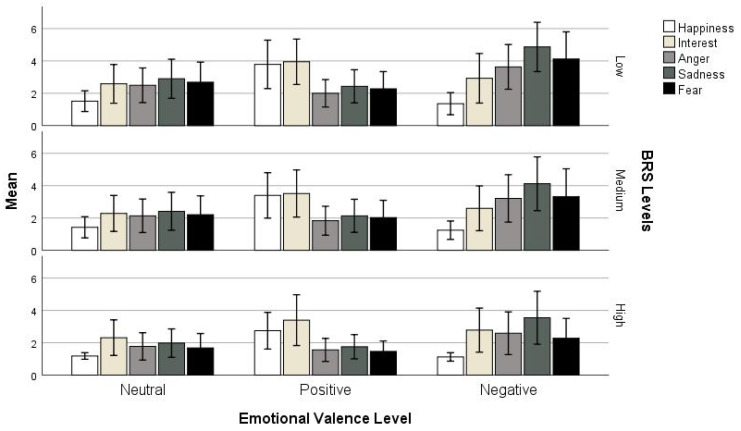
Mean ratings for emotional states across emotional valence levels for BRS levels, with error bars representing +/−1SD.

To test Hypothesis 3 and the effect of resilience on the emotional states evoked by newspaper headlines, a Repeated Measures ANOVA was conducted. Rating was treated as a dependent variable, emotional states (happiness, anger, sadness, fear, and interest) and emotional valence level (neutral, positive, and negative) as within-subjects factors, and BRS level as a fixed factor. A main effect of emotional states (*F*(4, 302) = 88.98, *p* < 0.001) and emotional valence level (*F*(2, 302) = 220.16, *p* < 0.001) was found, as well as strong significant interactions between emotional valence and BRS levels (*F*(4, 302) = 3.65, *p* < 0.01); emotional states and BRS levels (*F*(8, 302) = 4.12, *p* < 0.001); emotional valence and emotion (*F*(8, 302) = 65.27, *p* < 0.001); and emotional valence, emotional states, and BRS levels (*F*(16, 302) = 2.02, *p* < 0.02). Figure 2 shows the mean ratings for the five emotional states across emotional valence levels for the three BRS levels.

Univariate ANOVAs were run for each BRS level separately to examine the interaction between emotional states and BRS level, with rating as a dependent variable and emotional states as a fixed factor. Emotional states had a significant effect on rating in all BRS levels (low: *F*(4, 505) = 24.02, *p* < 0.001/medium: *F*(4, 840) = 25.02, *p* < 0.001/high: *F*(4, 125) = 5.82, *p* < 0.001). Post hoc tests revealed that all emotions differed between them in low and medium BRS levels, except anger, fear, and interest between them, with happiness being the lowest emotion and sadness the highest. In high BRS levels, interest differed from all emotional states except sadness; sadness differed from happiness and fear, and anger differed from interest. No other emotional states differed from each other. 

**Figure 3:**
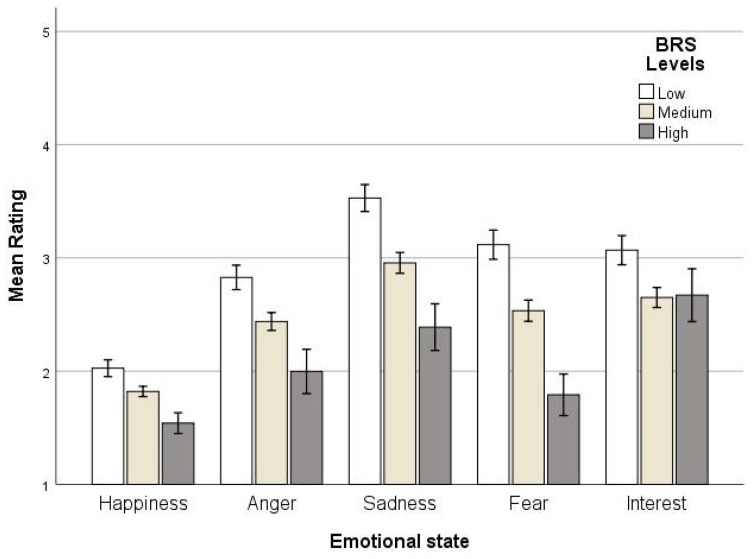
Mean ratings for emotional states for BRS levels, with error bars representing +/−1SD.

**Figure 4:**
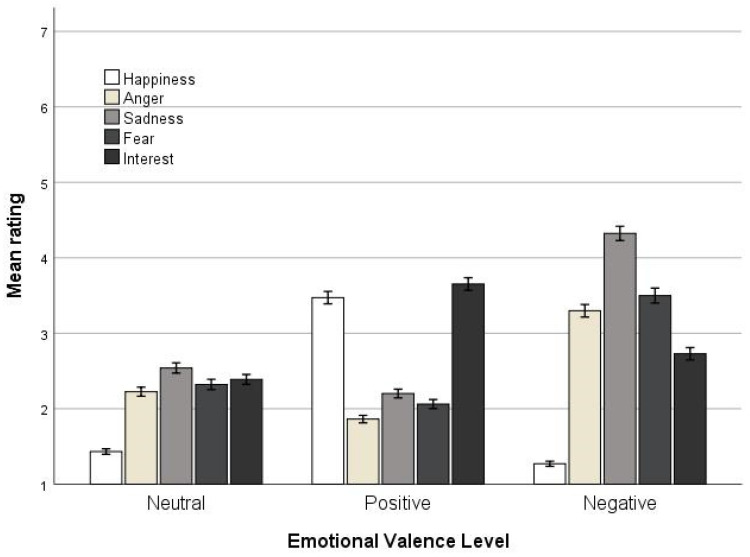
Mean ratings for emotional states across emotional valence level, with error bars representing +/−1SD.

To test how each emotional state differed across BRS levels, Univariate ANOVAs with rating and BRS level were conducted for the five emotional states separately. All ANOVAs showed a highly significant BRS level on rating (happiness: *F*(2, 294) = 6.74, *p* < 0.01/anger: *F*(2, 294) = 8.07, *p* < 0.001/sadness: *F*(2, 294) = 12.53, *p* < 0.001/fear: *F*(2, 294) = 14.25, *p* < 0.001/interest: *F*(2, 294) = 3.95, *p* < 0.05). Post hoc tests for happiness, anger, sadness, and fear showed that as BRS levels increased, emotion ranking fell (ps < 0.01). Finally, interest in medium BRS was lower than low BRS (*p* < 0.01). Figure 3 indicates the mean ratings for the five emotional states for the three BRS levels and Figure 4 the mean ratings for the five emotional states across emotional valence levels.

The interaction between emotional states and emotional valence levels was then examined. Univariate ANOVAs were run for each emotional valence level separately, with rating as a dependent variable and emotional state as a fixed factor. Emotional state had a significant effect on rating in all emotional valence levels (neutral: *F*(4, 1535) = 50.88, *p* < 0.001/positive: *F*(4, 1535) = 153.64, *p* < 0.001/negative: *F*(4, 1535) = 190.94, *p* < 0.001). Post hoc tests revealed that for neutral headlines, happiness was lower than all other emotional states (ps < 0.01), while sadness differed from anger and fear (*p* < 0.01). For positive headlines, happiness and interest received the highest ratings and did not differ from each other. Anger received the lowest ratings, while sadness and fear did not differ from each other. For negative headlines, all emotions differed from each other (ps < 0.01), except anger and fear, with happiness having the lowest ratings and sadness the highest. No other pairs were significantly different.

The three-way interaction between emotional states, BRS levels, and emotional valence levels was also further investigated, showing an overall drop in ratings as the BRS levels increased (see Figure 2). Univariate ANOVAs with emotional states (happiness, anger, sadness, fear, and interest) as a dependent variable and BRS levels as a fixed factor were run for each emotional valence level separately. For neutral headlines, all emotional states were shown to be affected by BRS levels (happiness: *F*(2, 307) = 3.20, *p* < 0.05/anger: *F*(2, 307) = 6.91, *p* < 0.01/sadness: *F*(2, 307) = 9.28, *p* < 0.01/fear: *F*(2, 307) = 10.03, *p* < 0.01), except interest (*F*(2, 307) = 2.33, *p* = 0.10). Post hoc tests showed that for all the emotional states that were shown to be affected by BRS levels, there was a significant drop in rating across BRS levels, with the highest levels showing lower emotional state ratings. Only happiness ratings did not differ between the low and medium BRS levels. For positive headlines, there was a significant effect of BRS levels on all emotional states (happiness: *F*(2, 307) = 6.53, *p* < 0.01/anger: *F*(2, 307) = 3.13, *p* < 0.05/sadness: *F*(2, 307) = 5.93, *p* < 0.01/fear: *F*(2, 307) = 6.93, *p* < 0.01/interest: *F*(2, 307) = 3.41, *p* < 0.05), with post hoc comparisons showing significant drops for happiness, sadness, and interest across BRS levels and a drop only in the high BRS group for anger and fear. Finally, for negative headlines, happiness and interest (happiness: *F*(2, 307) = 2.13, *p* = 0.12/interest: *F*(2, 307) = 1.72, *p* = 0.18) were not shown to be affected by BRS levels, but all other emotions showed a highly significant effect (anger: *F*(2, 307) = 6.66, *p* < 0.05/sadness: *F*(2, 307) = 10.60, *p* < 0.01/fear: *F*(2, 307) = 15.91, *p* < 0.01). Post hoc comparisons showed that these emotional state ratings dropped significantly as the BRS levels increased (ps < 0.01), except sadness ratings between medium and high BRS levels. 

## 4. Discussion

The current study contributes to the expanding body of literature on the effects of news headlines on individuals’ emotional states and overall well-being. The main aim of this study was to explore the emotions that individuals experience while reading headlines that are popular on social media news sites. Interestingly, while overall emotional reactions to headlines were not typically high, specific emotional responses such as negative emotions or heightened interest were noteworthy. This study also examined whether emotion regulation strategies and resilience would influence participants’ emotional experiences while reading the headlines and thus counterbalance the negative effects. As expected, we found that many participants reported experiencing negative emotions while reading the headlines, aligning with previous studies that linked news consumption, whether positive or negative, to influences on individuals’ well-being [34,52,53,54,55,56]. The results of the current study add to these findings by showing that headlines from news sites generating the most reactions in social media tend to elicit negative emotions in participants, with sadness being the predominant emotional response. Many participants also reported feeling fear and anger when reading the negative-valence headlines, with these two emotions not differing significantly from each other. The existing literature supports that anger and fear have some common elements, as both have negative valence and are motivated to avoid harm or failure [74]. The difference between the two is in their motivational direction; fear leads to withdrawal or escaping threats, whereas anger leads to actions to attack or remove obstacles [74]. Therefore, these common characteristics of anger and fear likely influenced our findings, shaping participants’ reactions to negative news headlines. This pattern of results underscores the potential of headlines alone, without the full story context, to profoundly influence emotional states. These findings are significant considering that repeated consumption of news with negative valence might negatively influence individuals’ well-being [34,52,53,54,56]. Thus, it can be inferred that individuals can experience negative emotions without the need to read the entire story; they can be emotionally influenced solely by reading the headline. These insights highlight the profound effect of headline news consumption on emotional health and suggest a critical area for further research into how social media news consumption influences individuals’ well-being.

Another important finding of the current study is that many participants reported experiencing interest in the headlines. According to the positive psychology literature, interest can be considered a positive emotion that involves being intrigued by something or an urge to explore or engage deeply with something [44,75]. Notably, interest, alongside happiness, was one of the highest-rated emotions when participants evaluated positive-valence headlines. This indicates that positive headlines are particularly effective at eliciting interest, suggesting that such content is engaging and motivates further exploration. Moreover, the current findings align with the existing literature indicating that interest and happiness are closely related, as both can be elicited by engaging and intriguing content [66]. However, our results also reveal a crucial distinction: while both happiness and interest are prominent in positive-valence headlines, interest remains high across headlines with neutral and negative valence. This contrast is particularly pronounced with negative-valence headlines, where there is a significant gap between the levels of happiness and interest expressed. This suggests that while the two emotions are related, they are distinct in their responsiveness to different emotional contexts. This is further supported by the preliminary analysis focusing on the overall emotion ratings (Table 1), which showed that across all emotional states examined, interest has the second-highest mean rating. Interest’s persistence across headlines, irrespective of their emotional valence, and particularly its robust presence in negative contexts, underscores its link to curiosity [76]. People may find stories or headlines interesting, irrespective of whether they evoke negative or positive emotions. The implication of the current findings is profound: interest plays a significant role in how individuals interact with media across diverse emotional contexts. The fact that interest remains high irrespective of the emotional valence of the headline suggests it is a crucial emotional state for new agencies to consider when aiming to capture and retain audience attention. Remarkably, there appears to be a gap in the literature regarding the impact of interest on individuals’ well-being while reading headlines or stories. Given the pervasive influence of interest observed in the current study, it is imperative for future research to further explore this emotion and its effects on audience engagement and well-being.

This study also examined whether emotion regulation strategies and resilience influence participants’ experience of emotions. As we expected, it was found that resilience influences the experience of emotions by the participants. The results showed that individuals with high resilience were less likely to experience both negative and positive emotions. It appears that resilience acts as a protective factor that shields individuals from experiencing strong emotions. This finding adds to the existing literature [48,62] by showing the importance of resilience in “protecting” one’s well-being. Regarding emotion regulation, it was hypothesized that these strategies would control the negative impact of headlines and, therefore, “protect” participants from experiencing negative emotions. Contrary to expectations, the results showed that emotion regulation was not related to the emotions reported by the participants. Even those who used these strategies still experienced heightened emotions (negative and positive). A possible explanation of this finding is that the strategies concerning emotion regulation might not have been actively engaged during this study. In the current study, we measured individuals’ emotion regulation strategies before presenting the headlines, which might have influenced the results. Additionally, the way we assessed individuals’ emotions focused more on emotional expression rather than emotional processing. This suggests that participants may not have applied these strategies while participating in the study. This finding underscores the potential benefit of incorporating emotional regulation strategies into news literacy training. By teaching individuals how to actively manage their emotional responses after encountering news with distressing or negative content, we can better equip them to minimize the influence of engagement with content with negative valence. Future research could explore this further by measuring emotion regulation strategies both prior to and after the presentation of the headlines.

This study was unique as it was the first study to directly examine the emotions that individuals experience when reading “popular” headlines from social media news sites. The findings contribute to the literature and create new avenues for future studies. However, this study was not without limitations. First, due to time constraints, participants were only asked to rate their emotional experience based on five predefined emotions. However, the literature on basic emotions suggests there are at least six [67,68], indicating that our study did not encompass the full spectrum potentially experienced by participants. Additionally, the lack of an option for participants to report emotions outside of those predefined restricts our ability to capture a broader range of emotional responses. Future research should consider examining a wider array of emotions and providing an open-ended option for participants to report any additional emotions they experience while reading the headlines. Second, the questionnaire that measured emotion regulation strategies was given prior to the presentation of the headlines, which might have influenced participants’ responses. Future studies can present the emotion regulation questionnaire after the questionnaire with the headlines or both prior and after. Third, to avoid potential biases associated with specific content categories, we have chosen to exclude headlines related to politics, religion, and sports. However, such content could potentially provide a more complete picture of the emotional experience of the participants, and this could be the focus of future research. Moreover, the gender imbalance in our sample, with more females than males, did not allow a more thorough examination of potential gender differences regarding emotional responses. Future research should aim to recruit a more balanced gender distribution for a more detailed examination of how emotional responses may vary between genders. Lastly, results obtained from self-report questionnaires should be viewed with caution. 

## 5. Conclusions

Social media have become deeply integrated into our lives, with many individuals reading daily news through these platforms. Often, this news is bombarded with negative information that can impact individuals’ well-being. This study contributes valuable insights into the emotional impact of social media headlines, demonstrating that these can influence emotional states profoundly, even without full engagement with the article content. Notably, our findings reveal that interest is a crucial factor in news consumption, impacting how individuals engage with and interact with news headlines of different emotional valence. The persistence of interests, especially in negative contexts, suggests that interest is driven by more than just the content’s positive appeal. It seems that it is a fundamental aspect of how individuals process and engage with information. Furthermore, the distinct emotional impacts of resilience, observed in the current study, suggest that fostering resilience may help mitigate the intensity of emotional responses to news.

The implications of these findings are significant. For researchers and news agencies, a deeper understanding of the role of interest and emotional engagement can guide more responsible and effective headline strategies, improving both the quality of media production and the public’s ability to interact with media content critically and healthily. For educators and mental health professionals, these results emphasize the need for enhanced news literacy training that includes strategies for managing the negative impact of news consumption. Clearly, our results suggest that regardless of their emotional valence (i.e., positive, negative, and neutral), headlines affect individuals’ emotional experiences.

Lastly, this study contributes to the literature by providing empirical evidence on the immediate emotional effects of engaging with popular news headlines from social media sites. It opens new avenues for future research to explore deeper into how these emotional responses impact long-term well-being and decision-making. 

## Figures and Tables

**Table 1:** Overall emotion ratings for the five emotional states.

	Happiness	Anger	Sadness	Fear	Interest
Rating	1.87 (0.66)	2.54 (1.06)	3.11 (1.22)	2.67 (1.28)	2.80 (1.22)

**Table 2:** Correlations between the five emotional states and age.

	Happiness	Interest	Anger	Sadness	Fear
Age	−0.006	0.085	0.039	0.011	0.005
Happiness		0.616 **	0.509 **	0.473 **	0.442 **
Interest			0.610 **	0.563 **	0.526 **
Anger				0.881 **	0.783 **
Sadness					0.835 **

Note. ** *p* < 0.01

## Data Availability

Data will be available upon reasonable request from the corresponding author.

## Appendix A

Table A1 depicts the headlines used in the current study, along with their mean and SD scores for each emotional state. Note that the original headlines were in the Greek language. Here, a translated English version is presented.

**Table A1:** Headlines together with mean and SD scores for emotional states.

Headline	Happiness Mean (SD)	Interest Mean (SD)	Anger Mean (SD)	Sadness Mean (SD)	Fear Mean (SD)
The young beaked whale that had washed ashore in Alimos has died.	1.07 (0.579)	2.56 (1.613)	2.50 (1.934)	4.09 (2.094)	2.60 (1.988)
2. Albania “does not recognize” degrees from Greek universities.	1.20 (0.876)	2.55 (1.764)	3.14 (2.133)	2.70 (2.017)	2.07 (1.745)
3. China records first case of H3N8 bird flu in a human.	1.12 (0.748)	3.14 (1.918)	2.43 (1.913)	3.02 (2.130)	4.08 (2.146)
4. Mpika Case: 27-year-old acquitted—Prosecutor: The accuser fabricated the story.	1.57 (1.347)	2.70 (1.808)	3.01 (2.141)	2.90 (2.174)	2.72 (2.046)
5. Video evidence: The moment the Albanian criminal is shot dead by police.	1.43 (1.159)	2.39 (1.679)	3.01 (2.141)	2.90 (2.174)	2.72 (2.046)
6. One of the four students who survived the Tempi tragedy committed suicide 19 years later.	1.43 (1.159)	2.39 (1.679)	2.83 (2.031)	3.19 (2.113)	3.10 (2.110)
7. NPHO: The first child’s death in Greece from acute hepatitis of unknown origin.	1.10 (0.615)	3.21 (1.923)	2.73 (2.042)	4.58 (2.147)	4.29 (2.095)
8. USA: Employee who hadn’t taken a day off in 27 years went viral—Strangers donated $300,000 to him.	2.98 (2.058)	3.05 (1.885)	1.62 (1.387)	1.69 (1.423)	1.67 (1.549)
9. Despina Vandi: Teared up singing “The Quiet Nights” 23 years after “Two Strangers”.	1.63 (1.328)	1.74 (1.388)	1.13 (0.717)	1.25 (0.879)	1.10 (0.610)
10. Woman gets paid to watch adult movies.	1.48 (1.256)	1.99 (1.574)	1.71 (1.499)	1.83 (1.615)	1.67 (1.455)
11. Makrinitsa: Double life sentence for the murderer of Konstantina and Giorgos.	2.91 (2.221)	3.17 (1.994)	2.70 (2.147)	2.90 (2.223)	2.77 (2.101)
12. Rafina: This is the 42-year-old man who was beaten to death—The fight and the fatal iron fist.	1.10 (0.644)	2.34 (1.642)	3.56 (2.206)	4.13 (2.224)	3.56 (2.233)
13. Weather now: “The heavens have opened” in Attica—Strong thunderstorm with lightning and thunder.	1.40 (1.041)	2.60 (1.747)	1.45 (1.273)	1.95 (1.615)	2.85 (1.979)
14. Heartbreaking farewell from the team of the 13-year-old who died in a soccer match in Evia.	1.07 (0.532)	2.59 (1.799)	2.28 (1.881)	4.86 (2.024)	3.15 (2.235)
15. Patras: The Ministry of Justice requests a disciplinary review of the forensic scientists for Malena and Irida.	2.29 (1.953)	3.52 (2.023)	3.04 (2.248)	3.18 (2.300)	2.88 (2.156)
16. Reggina Makedou: One of you saved me, one of you was 100% compatible with me.	4.64 (2.236)	4.10 (2.098)	1.17 (0.711)	1.39 (1.087)	1.73 (1.517)
17. Elafonisi, the “Caribbean” of Crete.	3.76 (2.245)	3.85 (2.148)	1.13 (0.675)	1.12 (0.636)	1.12 (0.687)
18. Egypt: Second deadly shark attack—Devoured a tourist from Romania.	1.13 (0.783)	2.58 (1.772)	2.19 (1.800)	4.08 (2.144)	3.36 (2.167)
19. Crete: Turnaround in an alimony case for the first time—The father was “released”.	1.44 (1.069)	2.67 (1.788)	2.12 (1.743)	2.12 (1.767)	2.03 (1.687)
20. Cinematic chase of a Roma gang in the northern suburbs.	1.23 (0.887)	2.33 (1.624)	2.44 (1.918)	2.39 (1.916)	2.93 (2.090)
21. Crowds flock to the Holy Church of Saints Isidores today despite the accusations of “false miracles”.	1.52 (1.312)	2.17 (1.663)	2.20 (1.859)	2.09 (1.760)	1.99 (1.738)
22. Russian influencers destroy Chanel bags and accuse the French house for “Russophobia”.	1.26 (0.889)	1.80 (1.333)	1.91 (1.640)	1.88 (1.635)	1.65 (1.457)
23. From Nafpaktos to… Harvard with a full scholarship, studying alone at home!	4.63 (2.266)	4.55 (2.042)	1.18 (0.836)	1.18 (0.850)	1.20 (0.819)
24. Spain: The bear that fell from a cliff is alive—It tried to save its cub.	3.35 (2.416)	3.64 (2.025)	1.32 (1.034)	2.70 (2.125)	1.86 (1.620)
25. Father beats priest at a school bus stop where he touched his 9-year-old son.	1.88 (1.768)	2.76 (1.867)	3.71 (2.227)	3.59 (2.275)	3.45 (2.186)
26. The family drama of Evridiki: My mother killed my father because he was raping me.	1.63 (1.446)	3.05 (1.909)	4.08 (2.306)	4.49 (2.248)	3.46 (2.265)
27. Hollywood stunned by Brad Pitt’s rare condition—He can’t even recognize his own family.	1.12 (0.717)	2.36 (1.549)	1.37 (1.114)	3.01 (2.023)	2.10 (1.702)
28. All her life they mocked her, calling her fat: The nightmare Gogo lived until her death at 14.	1.08 (0.588)	2.93 (1.912)	4.87 (2.073)	5.11 (1.990)	3.83 (2.318)
29. I slept with my landlord to support my children. I am ashamed, but I was forced to do it.	1.13 (0.666)	2.14 (1.584)	2.66 (2.062)	3.61 (2.237)	2.51 (2.044)
30. 35-year-old poured boiling water on her husband’s genitals because he dreamed of another woman in his sleep.	1.21 (0.899)	2.04 (1.545)	3.15 (2.218)	3.27 (2.170)	2.72 (2.140)
31. The father’s revenge: forced his friend and rapist of his 6-year-old daughter to dig his grave and buried him.	2.06 (1.857)	2.95 (1.930)	3.34 (2.228)	3.70 (2.287)	3.31 (2.205)
32. The whole village dressed in black: Unbearable pain at the funeral of the 16-year-old who drowned trying to retrieve a ball.	1.09 (0.652)	2.50 (1.720)	2.65 (2.070)	4.98 (2.011)	3.02 (2.218)
33. Tragic death of a 2-week-old baby—Hit by a car on its first walk with its parents.	1.08 (0.621)	2.59 (1.910)	3.66 (2.345)	5.38 (1.951)	3.58 (2.396)
34. Happy ending to the disappearance thriller of Thymios—found alive after 1.5 months.	4.42 (2.170)	3.78 (2.040)	1.61 (1.315)	1.99 (1.756)	2.07 (1.662)
35. Live Revenge: Father disguised and killed his 11-year-old son’s rapist in cold blood.	2.27 (1.905)	3.21 (1.955)	3.00 (2.199)	3.66 (2.279)	3.25 (2.200)
36. Her water broke at the beach: 37-year-old pregnant woman gives birth alone in the sea instead of going to the maternity hospital.	2.05 (1.608)	3.10 (1.845)	1.57 (1.313)	1.73 (1.449)	2.55 (1.875)
37. The three sisters took revenge: They killed their father because he raped them and made them dig their own pit.	2.21 (1.955)	3.19 (2.013)	3.86 (2.298)	4.23 (2.209)	3.23 (2.277)
38. Dead in a “pool of blood” wearing only underwear—tragedy with 25-year-old in Thessaloniki.	1.08 (0.616)	2.79 (1.878)	4.26 (2.311)	4.93 (2.050)	4.18 (2.264)
39. Her name is Maria, she was a “vegetable” living in the back wards at PAGNI, with countless health problems.	1.16 (0.768)	2.71 (1.728)	3.24 (2.261)	4.39 (2.154)	2.99 (2.190)
40. The murderer of 7-year-old Andreas was hanged with a sheet from the bed iron.	1.95 (1.732)	2.73 (1.926)	2.55 (2.047)	2.99 (2.207)	2.55 (2.055)
Total	1.85 (1.745)	2.81 (1.911)	2.56 (2.093)	3.13 (2.287)	2.70 (2.117)
